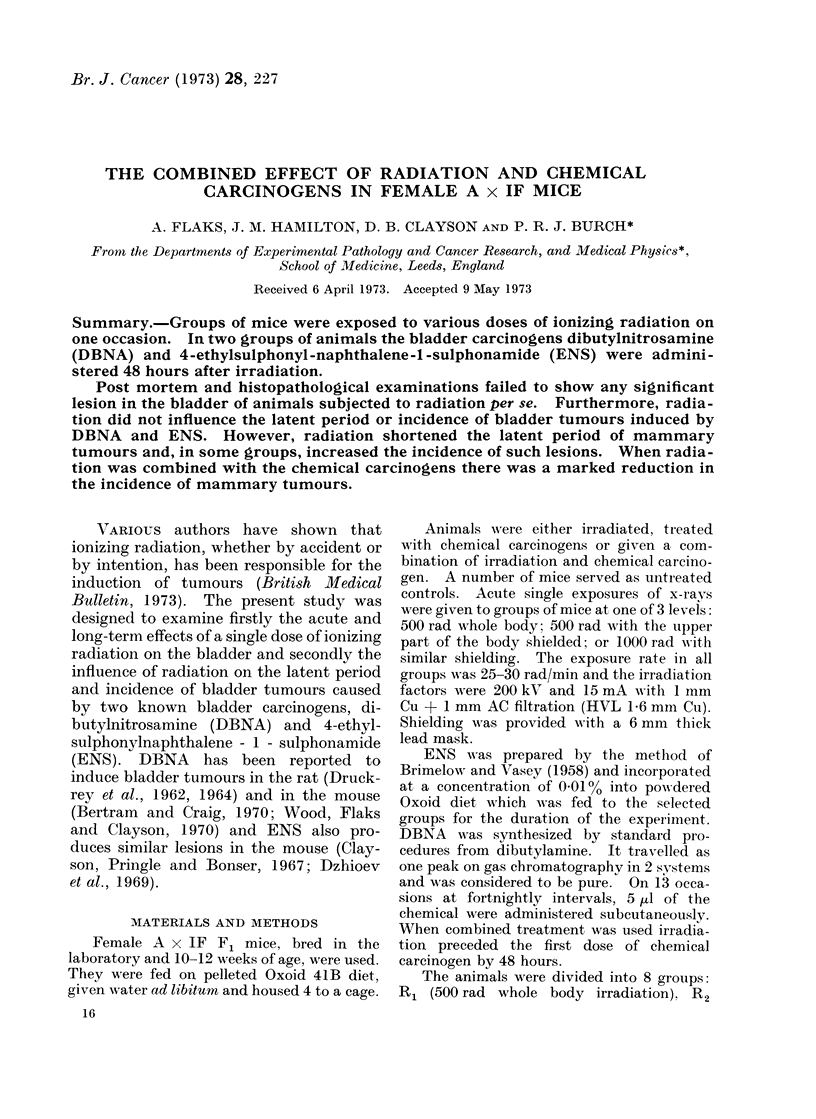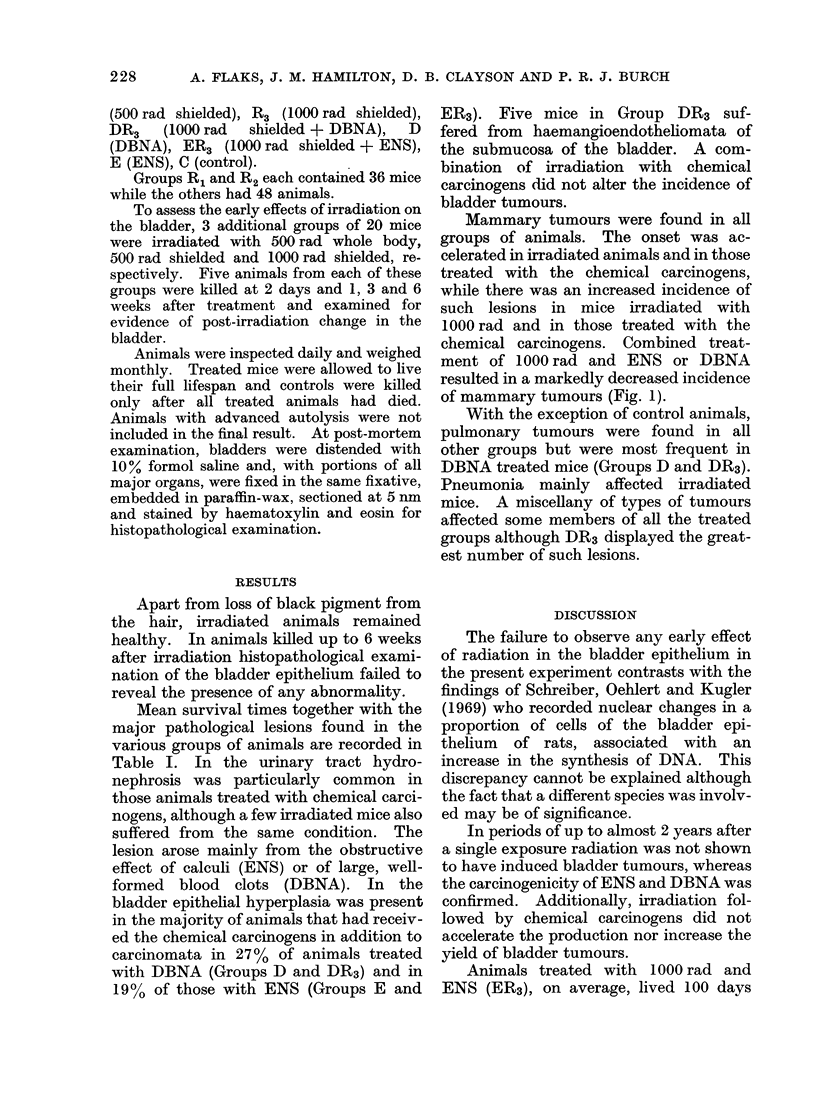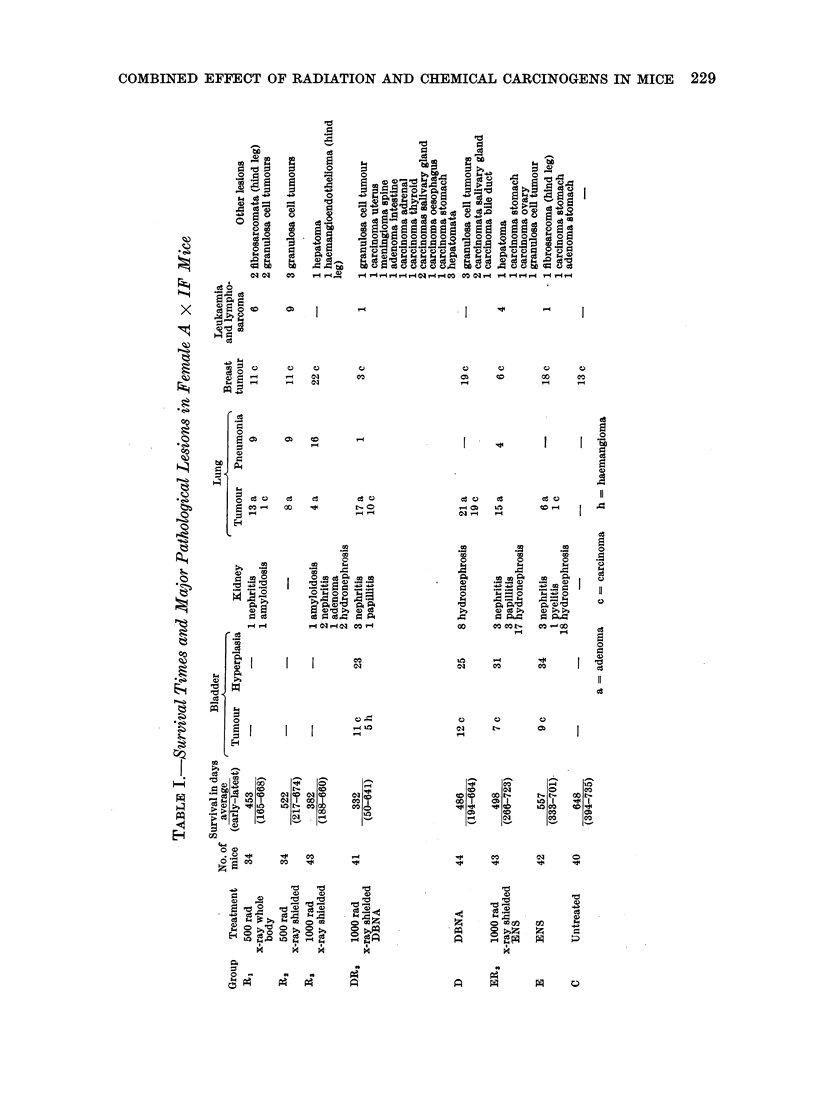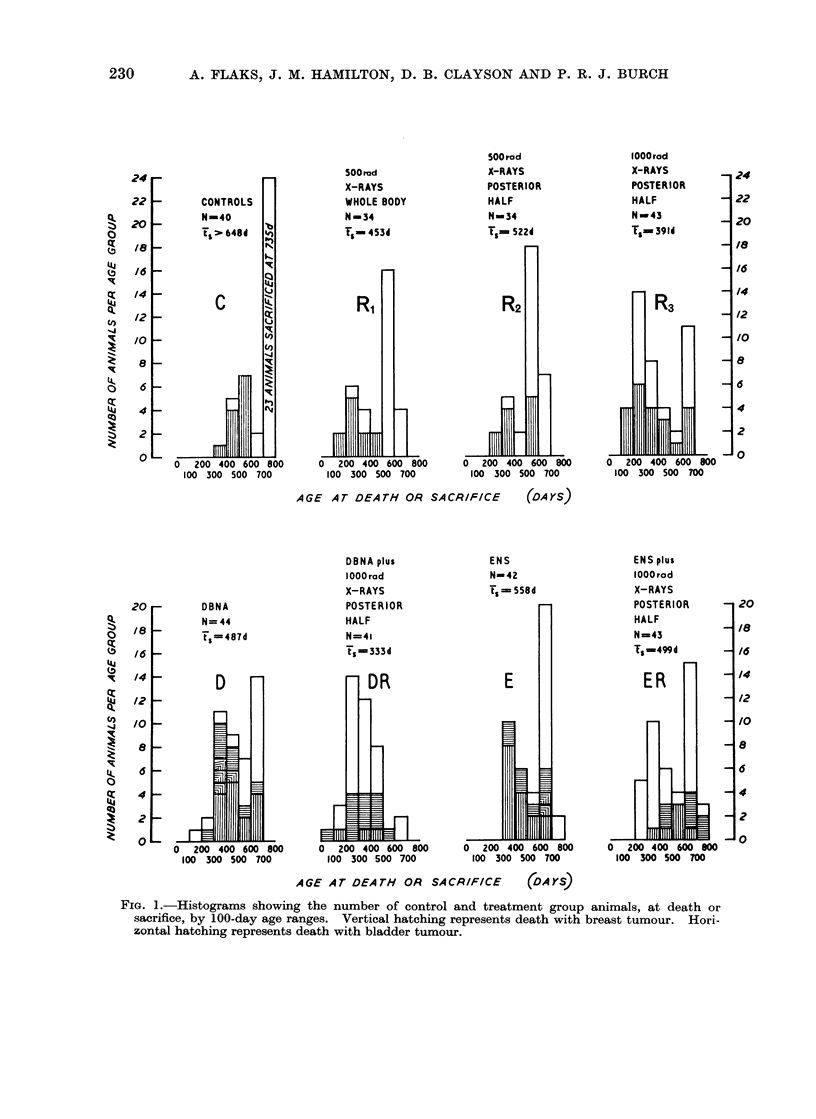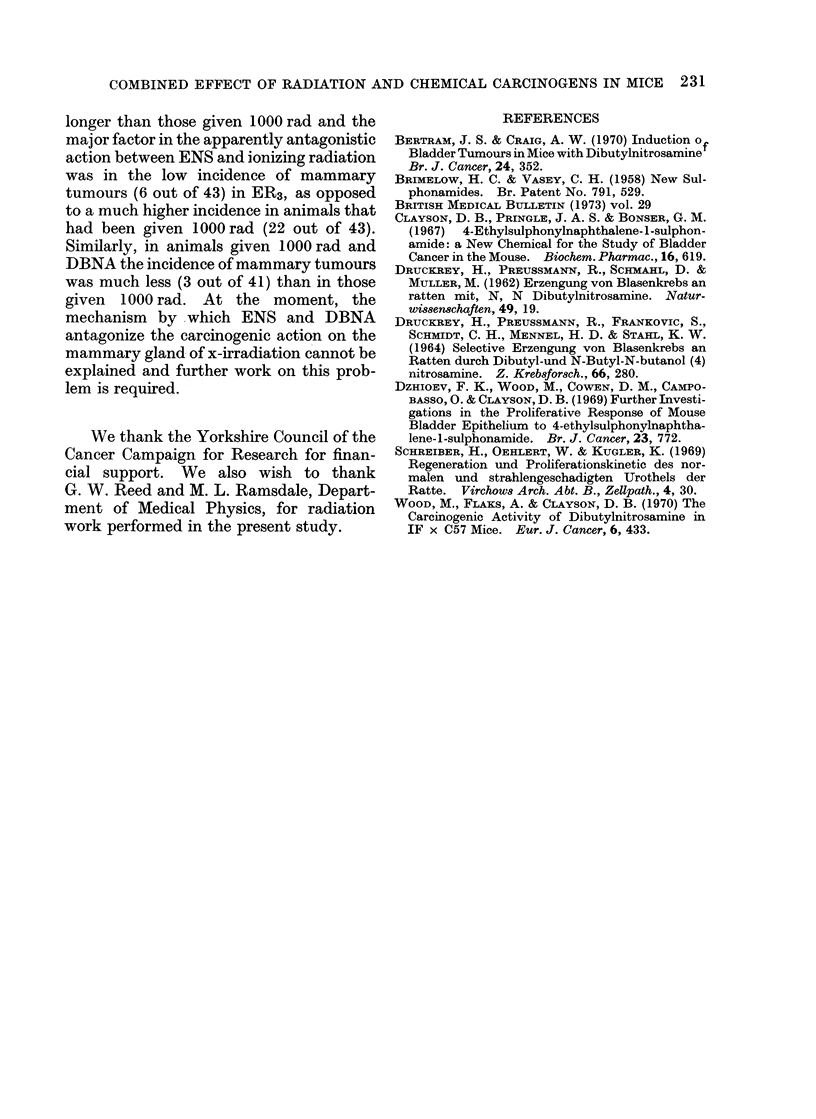# The Combined Effect of Radiation and Chemical Carcinogens in Female A × IF Mice

**DOI:** 10.1038/bjc.1973.141

**Published:** 1973-09

**Authors:** A. Flaks, J. M. Hamilton, D. B. Clayson, P. R. J. Burch

## Abstract

Groups of mice were exposed to various doses of ionizing radiation on one occasion. In two groups of animals the bladder carcinogens dibutylnitrosamine (DBNA) and 4-ethylsulphonyl-naphthalene-1-sulphonamide (ENS) were administered 48 hours after irradiation.

Post mortem and histopathological examinations failed to show any significant lesion in the bladder of animals subjected to radiation *per se.* Furthermore, radiation did not influence the latent period or incidence of bladder tumours induced by DBNA and ENS. However, radiation shortened the latent period of mammary tumours and, in some groups, increased the incidence of such lesions. When radiation was combined with the chemical carcinogens there was a marked reduction in the incidence of mammary tumours.


*Br. J. Cancer* (1973) **28**, 227

### THE COMBINED EFFECT OF RADIATION AND CHEMICAL CARCINOGENS IN FEMALE A × IF MICE

A. FLAKS, J. M. HAMILTON, D. B. CLAYSON AND P. R. J. BURCH*

*From the Departments of Experimental Pathology and Cancer Research, and Medical Physics*, School of Medicine, Leeds, England*

Received 6 April 1973. Accepted 9 May 1973

**Summary.—Groups of mice were exposed to various doses of ionizing radiation on one occasion. In two groups of animals the bladder carcinogens dibutylnitrosamine (DBNA) and 4-ethylsulphonyl-naphthalene-1-sulphonamide (ENS) were administered 48 hours after irradiation.**

**Post mortem and histopathological examinations failed to show any significant lesion in the bladder of animals subjected to radiation *per se*. Furthermore, radiation did not influence the latent period or incidence of bladder tumours induced by DBNA and ENS. However, radiation shortened the latent period of mammary tumours and, in some groups, increased the incidence of such lesions. When radiation was combined with the chemical carcinogens there was a marked reduction in the incidence of mammary tumours.**

VARIOUS authors have shown that ionizing radiation, whether by accident or by intention, has been responsible for the induction of tumours (*British Medical Bulletin*, 1973). The present study was designed to examine firstly the acute and long-term effects of a single dose of ionizing radiation on the bladder and secondly the influence of radiation on the latent period and incidence of bladder tumours caused by two known bladder carcinogens, dibutylnitrosamine (DBNA) and 4-ethylsulphonylnaphthalene - 1 - sulphonamide (ENS). DBNA has been reported to induce bladder tumours in the rat (Druckrey *et al.*, 1962, 1964) and in the mouse (Bertram and Craig, 1970; Wood, Flaks and Clayson, 1970) and ENS also produces similar lesions in the mouse (Clayson, Pringle and Bonser, 1967; Dzhioev *et al.*, 1969).

### MATERIALS AND METHODS

Female A × IF $F_1$ mice, bred in the laboratory and 10–12 weeks of age, were used. They were fed on pelleted Oxoid 41B diet, given water *ad libitum* and housed 4 to a cage.

Animals were either irradiated, treated with chemical carcinogens or given a combination of irradiation and chemical carcinogen. A number of mice served as untreated controls. Acute single exposures of x-rays were given to groups of mice at one of 3 levels: 500 rad whole body; 500 rad with the upper part of the body shielded; or 1000 rad with similar shielding. The exposure rate in all groups was 25–30 rad/min and the irradiation factors were 200 kV and 15 mA with 1 mm Cu + 1 mm AC filtration (HVL 1·6 mm Cu). Shielding was provided with a 6 mm thick lead mask.

ENS was prepared by the method of Brimelow and Vasey (1958) and incorporated at a concentration of 0·01% into powdered Oxoid diet which was fed to the selected groups for the duration of the experiment. DBNA was synthesized by standard procedures from dibutylamine. It travelled as one peak on gas chromatography in 2 systems and was considered to be pure. On 13 occasions at fortnightly intervals, 5 $\mu$l of the chemical were administered subcutaneously. When combined treatment was used irradiation preceded the first dose of chemical carcinogen by 48 hours.

The animals were divided into 8 groups: $R_1$ (500 rad whole body irradiation), $R_2$

(500 rad shielded), $R_3$ (1000 rad shielded), $DR_3$ (1000 rad shielded + DBNA), D (DBNA), $ER_3$ (1000 rad shielded + ENS), E (ENS), C (control).

Groups $R_1$ and $R_2$ each contained 36 mice while the others had 48 animals.

To assess the early effects of irradiation on the bladder, 3 additional groups of 20 mice were irradiated with 500 rad whole body, 500 rad shielded and 1000 rad shielded, respectively. Five animals from each of these groups were killed at 2 days and 1, 3 and 6 weeks after treatment and examined for evidence of post-irradiation change in the bladder.

Animals were inspected daily and weighed monthly. Treated mice were allowed to live their full lifespan and controls were killed only after all treated animals had died. Animals with advanced autolysis were not included in the final result. At post-mortem examination, bladders were distended with 10% formol saline and, with portions of all major organs, were fixed in the same fixative, embedded in paraffin-wax, sectioned at 5 nm and stained by haematoxylin and eosin for histopathological examination.

### RESULTS

Apart from loss of black pigment from the hair, irradiated animals remained healthy. In animals killed up to 6 weeks after irradiation histopathological examination of the bladder epithelium failed to reveal the presence of any abnormality.

Mean survival times together with the major pathological lesions found in the various groups of animals are recorded in Table I. In the urinary tract hydronephrosis was particularly common in those animals treated with chemical carcinogens, although a few irradiated mice also suffered from the same condition. The lesion arose mainly from the obstructive effect of calculi (ENS) or of large, well-formed blood clots (DBNA). In the bladder epithelial hyperplasia was present in the majority of animals that had received the chemical carcinogens in addition to carcinomata in 27% of animals treated with DBNA (Groups D and $DR_3$) and in 19% of those with ENS (Groups E and $ER_3$). Five mice in Group $DR_3$ suffered from haemangioendotheliomata of the submucosa of the bladder. A combination of irradiation with chemical carcinogens did not alter the incidence of bladder tumours.

Mammary tumours were found in all groups of animals. The onset was accelerated in irradiated animals and in those treated with the chemical carcinogens, while there was an increased incidence of such lesions in mice irradiated with 1000 rad and in those treated with the chemical carcinogens. Combined treatment of 1000 rad and ENS or DBNA resulted in a markedly decreased incidence of mammary tumours (Fig. 1).

With the exception of control animals, pulmonary tumours were found in all other groups but were most frequent in DBNA treated mice (Groups D and $DR_3$). Pneumonia mainly affected irradiated mice. A miscellany of types of tumours affected some members of all the treated groups although $DR_3$ displayed the greatest number of such lesions.

### DISCUSSION

The failure to observe any early effect of radiation in the bladder epithelium in the present experiment contrasts with the findings of Schreiber, Oehlert and Kugler (1969) who recorded nuclear changes in a proportion of cells of the bladder epithelium of rats, associated with an increase in the synthesis of DNA. This discrepancy cannot be explained although the fact that a different species was involved may be of significance.

In periods of up to almost 2 years after a single exposure radiation was not shown to have induced bladder tumours, whereas the carcinogenicity of ENS and DBNA was confirmed. Additionally, irradiation followed by chemical carcinogens did not accelerate the production nor increase the yield of bladder tumours.

Animals treated with 1000 rad and ENS ($ER_3$), on average, lived 100 days

TABLE I.—*Survival Times and Major Pathological Lesions in Female A × IF Mice*

| Group | Treatment | No. of mice | Survival in days average (early–latest) | Bladder | | Kidney | Lung | | Breast tumour | Leukaemia and lympho-sarcoma | Other lesions |
|---|---|---|---|---|---|---|---|---|---|---|---|
| | | | | Tumour | Hyperplasia | | Tumour | Pneumonia | | | |
| $R_1$ | 500 rad x-ray whole body | 34 | 453 (165–668) | — | — | 1 nephritis<br>1 amyloidosis | 13 a<br>1 c | 9 | 11 c | 6 | 2 fibrosarcomata (hind leg)<br>2 granulosa cell tumours |
| $R_2$ | 500 rad x-ray shielded | 34 | 522 (217–674) | — | — | — | 8 a | 9 | 11 c | 9 | 3 granulosa cell tumours |
| $R_3$ | 1000 rad x-ray shielded | 43 | 382 (188–660) | — | — | 1 amyloidosis<br>2 nephritis<br>1 adenoma<br>2 hydronephrosis | 4 a | 16 | 22 c | — | 1 hepatoma<br>1 haemangioendothelioma (hind leg) |
| $DR_3$ | 1000 rad x-ray shielded DBNA | 41 | 332 (50–641) | 11 c<br>5 h | 23 | 3 nephritis<br>1 papillitis | 17 a<br>10 c | 1 | 3 c | 1 | 1 granulosa cell tumour<br>1 carcinoma uterus<br>1 meningioma spine<br>1 adenoma intestine<br>1 carcinoma adrenal<br>1 carcinoma thyroid<br>2 carcinomas salivary gland<br>1 carcinoma oesophagus<br>1 carcinoma stomach<br>3 hepatomata |
| D | DBNA | 44 | 486 (194–664) | 12 c | 25 | 8 hydronephrosis | 21 a<br>19 c | — | 19 c | — | 3 granulosa cell tumours<br>2 carcinomata salivary gland<br>1 carcinoma bile duct |
| $ER_3$ | 1000 rad x-ray shielded ENS | 43 | 498 (266–723) | 7 c | 31 | 3 nephritis<br>3 papillitis<br>17 hydronephrosis | 15 a | 4 | 6 c | 4 | 1 hepatoma<br>1 carcinoma stomach<br>1 carcinoma ovary<br>1 granulosa cell tumour |
| E | ENS | 42 | 557 (333–701) | 9 c | 34 | 3 nephritis<br>1 pyelitis<br>18 hydronephrosis | 6 a<br>1 c | — | 18 c | 1 | 1 fibrosarcoma (hind leg)<br>1 carcinoma stomach<br>1 adenoma stomach |
| C | Untreated | 40 | 648 (394–735) | — | — | — | — | — | 13 c | — | — |

a = adenoma c = carcinoma h = haemangioma

FIG. 1.—Histograms showing the number of control and treatment group animals, at death or sacrifice, by 100-day age ranges. Vertical hatching represents death with breast tumour. Horizontal hatching represents death with bladder tumour.

longer than those given 1000 rad and the major factor in the apparently antagonistic action between ENS and ionizing radiation was in the low incidence of mammary tumours (6 out of 43) in $ER_3$, as opposed to a much higher incidence in animals that had been given 1000 rad (22 out of 43). Similarly, in animals given 1000 rad and DBNA the incidence of mammary tumours was much less (3 out of 41) than in those given 1000 rad. At the moment, the mechanism by which ENS and DBNA antagonize the carcinogenic action on the mammary gland of x-irradiation cannot be explained and further work on this problem is required.

We thank the Yorkshire Council of the Cancer Campaign for Research for financial support. We also wish to thank G. W. Reed and M. L. Ramsdale, Department of Medical Physics, for radiation work performed in the present study.

### REFERENCES

BERTRAM, J. S. & CRAIG, A. W. (1970) Induction of Bladder Tumours in Mice with Dibutylnitrosamine *Br. J. Cancer*, **24**, 352.

BRIMELOW, H. C. & VASEY, C. H. (1958) New Sulphonamides. Br. Patent No. 791, 529.

BRITISH MEDICAL BULLETIN (1973) vol. 29

CLAYSON, D. B., PRINGLE, J. A. S. & BONSER, G. M. (1967) 4-Ethylsulphonylnaphthalene-1-sulphonamide: a New Chemical for the Study of Bladder Cancer in the Mouse. *Biochem. Pharmac.*, **16**, 619.

DRUCKREY, H., PREUSSMANN, R., SCHMAHL, D. & MULLER, M. (1962) Erzengung von Blasenkrebs an ratten mit, N, N Dibutylnitrosamine. *Naturwissenschaften*, **49**, 19.

DRUCKREY, H., PREUSSMANN, R., FRANKOVIC, S., SCHMIDT, C. H., MENNEL, H. D. & STAHL, K. W. (1964) Selective Erzengung von Blasenkrebs an Ratten durch Dibutyl-und N-Butyl-N-butanol (4) nitrosamine. *Z. Krebsforsch.*, **66**, 280.

DZHIOEV, F. K., WOOD, M., COWEN, D. M., CAMPOBASSO, O. & CLAYSON, D. B. (1969) Further Investigations in the Proliferative Response of Mouse Bladder Epithelium to 4-ethylsulphonylnaphthalene-1-sulphonamide. *Br. J. Cancer*, **23**, 772.

SCHREIBER, H., OEHLERT, W. & KUGLER, K. (1969) Regeneration und Proliferationskinetic des normalen und strahlengeschadigten Urothels der Ratte. *Virchows Arch. Abt. B., Zellpath.*, **4**, 30.

WOOD, M., FLAKS, A. & CLAYSON, D. B. (1970) The Carcinogenic Activity of Dibutylnitrosamine in IF × C57 Mice. *Eur. J. Cancer*, **6**, 433.